# {2-[1-(3-Methoxycarbonylmethyl-1H-indol-2-yl)-1-methyl-ethyl]-1H-indol-3-yl}-acetic Acid Methyl Ester Inhibited Hepatocellular Carcinoma Growth in Bel-7402 Cells and Its Resistant Variants by Activation of NOX4 and SIRT3

**DOI:** 10.1155/2015/491205

**Published:** 2015-04-15

**Authors:** Ye Li, Wenjing Wang, Xiaoxue Xu, Shiyue Sun, Xiaoyu Xu, Xian-jun Qu

**Affiliations:** ^1^Department of Pharmacology, School of Chemical Biology & Pharmaceutical Sciences, Capital Medical University, Beijing 100069, China; ^2^Department of Medicinal Chemistry, School of Chemical Biology & Pharmaceutical Sciences, Capital Medical University, Beijing 100069, China; ^3^Medical Experiments and Testing Center, Capital Medical University, Beijing 100069, China

## Abstract

{2-[1-(3-Methoxycarbonylmethyl-1H-indol-2-yl)-1-methyl-ethyl]-1H-indol-3-yl}-acetic acid methyl ester (MIAM) is a novel indole compound, which possessed high efficacy against many cancers xenografted in mice without obvious toxicity. In this study, we aimed to investigate the effects of MIAM on human hepatocellular carcinoma (HCC) Bel-7402 cells and its resistant variants Bel-7402/5FU. MIAM inhibited the growth of HCC more potent in Bel-7402/5FU cells than its parent cells. MIAM increased cellular reactive oxygen species (ROS) levels, induced cell apoptosis, and arrested cell cycle in G_0_/G_1_ phase. MIAM might exert its action on Bel-7402/5FU cells through activation of NADPH oxidase 4 (NOX4)/p22^phox^, Sirtuin3 (SIRT3)/SOD2, and SIRT3/p53/p21^Waf1/Cip^ pathways. MIAM might inhibit HCC growth through the modulation of SIRT3. When SIRT3 was silenced, the inhibitory effect of MIAM on Bel-7402/5FU was lowered, showing the characteristic of resistance against MIAM, whereas Bel-7402/5FU cells with high expression of SIRT3 by SIRT3 adenovirus infection demonstrated the high sensitivity to MIAM. These results suggested that MIAM might exert its action against Bel-7402/5FU growth through upregulation of SIRT3. We suggested that MIAM might be a promising candidate compound which could develop as a potent anticancer agent targeting NOX4 and SIRT3 activation.

## 1. Introduction

Hepatocellular carcinoma (HCC) is one of the most lethal malignancies due to difficulty of early detection and chemoresistance [1]. HCC is characterized by the intrinsic and acquired resistance to available chemotherapeutic agents and eventually results in poor prognosis in patients. Although many efforts have been made, significant improvement in chemotherapy has not been achieved [2, 3].

Sirtuin3 (SIRT3) is the NAD^+^-dependent deacetylases localized in mitochondria. SIRT3 has been found to play important roles in maintaining mitochondrial function and integrity in response to the oxidative stress. SIRT3 involves in metabolism, ATP generation, and oxidative stress by deacetylasing lysine residues of mitochondrial proteins [4, 5]. High expression of SIRT3 has been considered to suppress HCC growth, invasion, and acquired resistance [6, 7]. Low level of SIRT3 was associated with poor differentiation and progression of HCC [8–10]. For example, deletion of SIRT3 in mouse embryonic fibroblasts exhibited the phenotype of high proliferation, antiapoptosis, and the characteristic of invasion and metastasis [11]. Cancer cells with deletion of SIRT3 might result in antiapoptotic phenotype through the mechanism of preventing the Bak- or Bax-induced mitochondrial damage [12, 13]. SIRT3 has thus been considered to be an important target for design and use of chemotherapeutic drugs.

{2-[1-(3-Methoxycarbonylmethyl-1H-indol-2-yl)-1-methyl-ethyl]-1H-indol-3-yl}-acetic acid methyl ester (MIAM) is an analogue of 3,3′-diindolylmethane, which has demonstrated DNA intercalating and topoisomerase inhibitory effects* in vitro*. Previous study showed that MIAM possessed strong activity against the growth of many types of cancers xenografted in mice. In S180 sarcoma mouse model, MIAM with 0.45 mg/kg, 4.5 mg/kg, and 45 mg/kg by injection inhibited cancer growth by 19.5%, 40.7%, and 67.8%, respectively [14]. MIAM did not show significant toxicity to mice when orally administrated at the daily dose of 400 mg/kg. However, the effects of this compound have required the continued determination and the mechanism of MIAM action has not been fully revealed. In this study, we aimed to determine the effects of MIAM on HCC cell line Bel-7402 and its 5-fluorouracil resistant variant Bel-7402/5FU. The mechanism of MIAM action on these cell lines was investigated.

## 2. Materials and Methods

### 2.1. Cell Lines and Culture

Human HCC cell line Bel-7402 was purchased from cell bank of Chinese Academy of Sciences (Shanghai). Cells were cultured in RPMI-1640 medium supplemented with 10% (v/v) fetal bovine serum and penicillin-streptomycin (100 IU/mL–100 *μ*g/mL) at 37°C in a humidified atmosphere (5% CO_2_-95% air). Bel-7402/5FU, a 5-fluorouracil resistant variant, was obtained from The Institute of Pharmaceutical Science Research, Chinese Academy of Sciences (Beijing). The biological characteristics of Bel-7402/5FU were described previously [15]. Bel-7402/5FU cells were maintained in the medium containing 20 mg/L of 5-fluorouracil (F6627, Sigma). The cells were cultured without 5-fluorouracil for two weeks before experiments.

### 2.2. Chemicals and Treatment Methods

MIAM was designed and synthesized as described previously [14]. The purity of compound as measured by high performance liquid chromatography (HPLC) was 98.5%. Cancer cells were treated either with MIAM, control drug adriamycin (ADR, D1515, Sigma), or equal volume of vehicle (DMSO) for 24, 48, and 72 h, respectively. Cell viability was estimated by the 3-[4,5-dimethylthiazol-2-yl]-2,5-diphenyltetrazolium bromide (MTT, Sigma) assay as described elsewhere. Briefly, hepatoma cells were seeded in 96-well plates at a density of 4 × 10^3^ cells/well and incubated at 37°C in 5% CO_2_ for 24 h. After 48 h incubation, MTT assay was performed by adding 20 *μ*L of MTT (5 mg/mL) to each well and incubated for 4 h at 37°C. The absorbance at 492 nm was measured using a microplate reader. Cell viability in experimental groups was estimated by comparing to vehicle control.

### 2.3. Assay of Intercellular Reactive Oxygen Species (ROS) Levels

The level of cellular ROS was determined by using high content screening assay based on the mechanism of oxidation of 2′,7′-dichlorofluorescin diacetate (DCFH-DA) to fluorescent 2′,7′-dichlorofluorescin (DCF) which is incorporated into cells (excitation/emission 488/530 nm). Hoechst 33342 (excitation/emission 350/461 nm) stains DNA in the living cells. Cancer cells seeded in 96-well plate were incubated with different concentrations of MIAM for 72 h. After washing, cells were incubated with serum-free medium containing 10 *μ*M of DCFH-DA in the dark for 20 min at 37°C. Cells were washed and incubated with serum-free medium containing 0.5 *μ*g/mL of Hoechst 33342. The images of cells were acquired in an automated manner by ArrayScan XTI high-content imaging instrumentation (Thermo Fisher). Cells with green fluorescence were determined to be the product of ROS. The increase of ROS was determined based on the average percent of cells with green fluorescence [16].

### 2.4. Annexin V/FITC/PI Staining Assay

Cancer cells seeded in 6-well plates (2 × 10^5^ cells/well) were exposed to different concentrations of MIAM for 72 h. Control cells were exposed to an equal volume of vehicle. Cells were harvested and washed with cold PBS. The levels of phosphatidylserine in the surface of apoptotic cells were quantitatively determined by using the Annexin-V/FITC and PI apoptosis detection kit. The analysis of apoptotic cells was performed on a FACScan flow cytometry (Becton-Dickinson).

### 2.5. Cell Cycle Analysis

Cancer cells (2 × 10^5^ cells/well) treated with MIAM were harvested, washed, and fixed in cold 90% ethanol for 30 min. Cells were collected and suspended in 1 mL PI solution (20 mg/mL PI (Sigma), 0.2 mg/mL DNase-free RNase A, and 0.1% triton X-100 in PBS) for 30 min. Cell cycle was analyzed by FACScan flow cytometer (Becton-Dickinson). The population of cells in G_0_/G_1_, S, and G_2_/M phases was determined by using ModFit LT software 3.0 (Varity Software House, Topsham).

### 2.6. Western Blotting Analysis

Cancer cells treated with MIAM were lysed in buffer containing 50 mM HEPES (pH 7.4), 150 mM NaCl, 0.1% triton X-100, 1.5 mM MgCl_2_, 1 mM EDTA, 2 mM sodium orthovanadate, 4 mM sodium pyrophosphate, 100 mM NaF, and protease inhibitor mixture (Roche) on ice. Equal amounts of proteins were separated by SDS-PAGE electrophoresis and then electrotransferred onto PVDF membrane, blocked with 5% nonfat dry milk for 1 h at room temperature. The levels of proteins were determined by incubation with primary antibodies with appropriate dilution overnight. The primary antibodies included those specific to Bax (sc-493), Bcl-2 (sc-492), p53 (sc-126), phospho-Rb (Ser 795) (p-Rb, sc-21875), p21^Waf1/Cip1^ (sc-397), p22^phox^ (sc-20781), BIM (sc-11425), *β*-actin (sc-47778, Santa Cruz), NOX4 (14347-1-AP, ProteinTech), and SIRT3 (#2627, Cell Signaling). The bound antibodies were visualized by using an enhanced chemiluminescence reagent (Millipore) and quantified by densitometry using ChemiDoc XRS+ image analyzer (Bio-Rad).

### 2.7. Measurement of Mitochondrial Membrane Potential

The fluorescent, lipophilic, and cationic probe, JC-1 (Beyotime, China), was used to measure the mitochondrial membrane potential (ΔΨm) of cancer cells according to the manufacturer's instruction which has been described previously [17]. Briefly, cells were plated in 12-well plates and treated with different concentrations of MIAM for 72 h. Cells were loaded with JC-1 for 30 min at 37°C. Green and red fluorescence intensities were detected using an infinite M200 microplate reader. The ΔΨm of cancer cells in each treatment group was calculated as the fluorescence ratio of red to green and was expressed as a multiple of the level in the vehicle group. As a positive control, mitochondria were depolarized by incubating cells with 50 *μ*M carbonylcyanidem-chlorophenyl-hydrazone (CCCP) for 5 min at 37°C. For JC-1 green, Ex = 485 nm and Em = 525 nm; for JC-1 red, Ex = 535 nm and Em = 590 nm.

### 2.8. Transfection of SIRT3-Targeted siRNA or Adenovirus Expressing SIRT3

Cancer cells grown in the exponential phase were seeded in 96-well plate and were then transfected with a SIRT3-targeted siRNA (sc-61555, Santa Cruz) for 6 h or nontargeting RNA by using Lipofectamine RNAiMax and Opti-MEM reduced serum medium (Invitrogen), according to the manufacturer's instructions. For virus transfection, cells were exposed to human SIRT3 adenovirus (Hanbio Biotechnology, China) for 2 h. The titer of virus was 1 × 10^10^ plaque formation units (PFU)/mL as measured by plaque assays before experiments. The levels of SIRT3 were determined by Western blotting assay.

### 2.9. Statistical Analysis

Data are presented as means ± S.D. and were analyzed by one-way ANOVA. A *P* value < 0.05 was considered statistically significant. Statistical analysis was performed using SPSS/Win13.0 software (SPSS, Inc., Chicago, Illinois).

## 3. Results and Discussion

### 3.1. MIAM Inhibited HCC Growth More Profoundly in Bel-7402/5FU Cells Than Its Parent Cells

**Figure 1:**
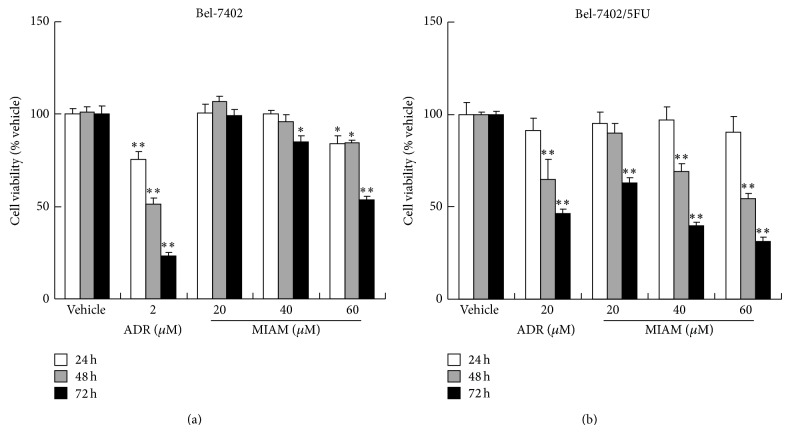
MIAM inhibited the proliferation of Bel-7402 and Bel-7402/5FU cells* in vitro*. HCC cells seeded in the 96-well plates were exposed to MIAM and ADR for up to 72 h. Viable cells were estimated by the MTT assay and denoted as a percentage of untreated controls at the concurrent time point. The bars indicate mean ± S.D. (*n* = 5). ^∗^ *P* < 0.05, ^∗∗^ *P* < 0.01 versus the vehicle control.

It is well known that HCC is less sensitive to most chemotherapeutic agents for the frequent de novo and acquired chemoresistance. Bel-7402/5FU cells are drug resistant HCC cells against multiple agents including 5-fluorouracil and ADR [15]. In our previous study, we suggested that MIAM might inhibit cancer growth through intercalating to DNA suppressing topoisomerase activity like ADR [14]. In this study, we therefore selected ADR as the positive control. As shown in Figure 1(a), ADR strongly inhibited Bel-7402 growth. ADR with 2 *μ*M significantly inhibited Bel-7402 growth by 75.6% (*P* < 0.01 versus the vehicle control), whereas ADR with this concentration did not significantly affect the growth of Bel-7402/5FU. We used high concentration of ADR exposure to Bel-7402/5FU. As shown in Figure 1(b), when ADR was reached by up to 20 *μ*M for 72 h, the reduction of Bel-7402/5FU growth was seen with percent of inhibition by 53.7% (Figure 1(b), *P* < 0.01 versus the vehicle control).

MIAM presented the activity against the growth of HCC in both Bel-7402 and Bel-7402/5FU cells. As shown in Figure 1(a), MIAM prevented HCC growth with a time-dependent manner. In the concentrations ranging from 20 to 60 *μ*M for 72 h, the percent of inhibition on Bel-7402 cells was 1.7% (*P* > 0.05 versus the vehicle control), 15.2% (*P* > 0.05 versus the vehicle control), and 46.2% (*P* < 0.01 versus the vehicle control), respectively. In contrast, Bel-7402/5FU cells were shown to be more sensitive to MIAM than Bel-7402 cells. As shown in Figure 1(b), MIAM with 20, 40, and 60 *μ*M for 72 h significantly prevented Bel-7402/5FU growth by 37.1% (*P* < 0.01 versus the vehicle control), 60.1% (*P* < 0.01 versus the vehicle control), and 68.7% (*P* < 0.01 versus the vehicle control), respectively. A significant difference was observed between Bel-7402/5FU and Bel-7402 (*P* < 0.05). These results indicated that MIAM inhibited HCC growth more potential in Bel-7402/5FU cells than its parent cells.

### 3.2. MIAM Induced HCC Apoptosis in Bel-7402/5FU Cells by Induction of Mitochondrial Membrane Potential Collapse and Increases of Bax/Bcl-2 Ratio

**Figure 2:**
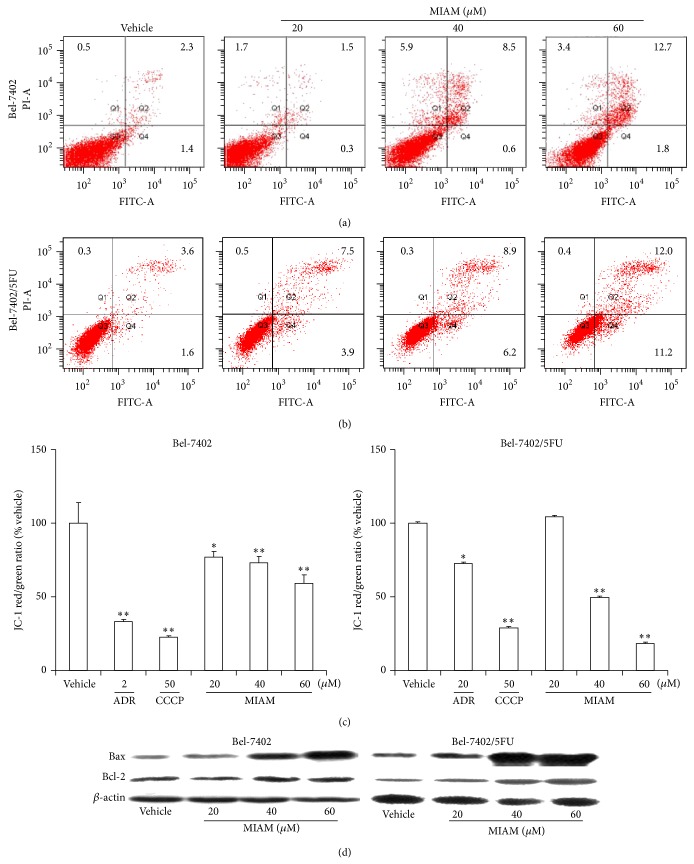
MIAM induced apoptosis in HCC cells. (a-b) Cells were exposed to different concentrations of MIAM for 72 h and then the percent of apoptotic cells were determined by using flow cytometry assay. (c) MIAM induced mitochondrial membrane potential collapse in Bel-7402 and Bel-7402/5FU cells as observed by JC-1 staining. The bars indicate mean ± S.D. (*n* = 5). ^∗^ *P* < 0.05, ^∗∗^ *P* < 0.01 versus the vehicle control. (d) MIAM regulated Bax and Bcl-2 expressions in Bel-7402 and Bel-7402/5FU cells. Data are representative of 3 independent experiments.

The acquired resistant nature of Bel-7402/5FU cells is characterized by the dysregulation of cancer behaviors governing cell proliferation and survival, among which the noteworthy are the oxidative stress status and apoptosis resistance [18]. In this study, MIAM showed the activity of induction apoptosis in both Bel-7402/5FU and Bel-7402 cells. However, Bel-7402/5FU cells were more sensitive than its parent Bel-7402 cells to MIAM. As shown in Figure 2, MIAM at concentrations of 20, 40, and 60 *μ*M significantly induced the percent of apoptotic cells in Bel-7402 by 9.1%, 14.5%, and 12.7% (Figure 2(a), *P* < 0.05 versus the vehicle control), respectively, and in Bel-7402/5FU by 11.4%, 15.1%, and 22.2% (Figure 2(b), *P* < 0.05 versus the vehicle control), respectively.

We used JC-1 fluorescence probe to show the mitochondrial membrane potential in HCC cells. As shown in Figure 2(c), MIAM at the concentrations of 40 and 60 *μ*M resulted in the decrease of red to green fluorescence in Bel-7402/5FU by 50.3% and 81.7% (Figure 2(c), left, *P* < 0.01 versus the vehicle control), respectively, and in Bel-7402 by 27.2% and 41.0% (Figure 2(c), right, *P* < 0.01 versus the vehicle control), respectively. The result suggested that MIAM might induce HCC cells to apoptosis through the mechanism of mitochondrial disruption.

The process of apoptosis initiated by mitochondrial membrane damage is carefully controlled by the Bcl-2 protein family and requires the activation of proapoptotic members such as p53 and Bax [19]. Western blotting analysis could determine different changes of the apoptotic proteins in HCC. As shown in Figure 2(d), MIAM significantly increased the levels of Bax and Bcl-2 in both Bel-7402 and Bel-7402/5FU cells. Statistical analysis revealed that the ratios of Bax/Bcl-2 in Bel-7402 cells were from 0.29 in the control cells to 0.54, 2.17, and 2.93, respectively, and in Bel-7402/5FU cells from 1.54 in the control cells to 1.76, 6.21, and 6.45, respectively. A significant difference existed between Bel-7402/5FU and Bel-7402 cells (*P* < 0.05). These results suggested that MIAM might induce Bel-7402/5FU cells to apoptosis through inducing mitochondrial membrane potential collapse and increasing the ratio of Bax/Bcl-2.

### 3.3. MIAM Treatment Resulted in Cell Cycle Arrest in G_0_/G_1_ Phase in Bel-7402/5FU Cells

**Figure 3:**
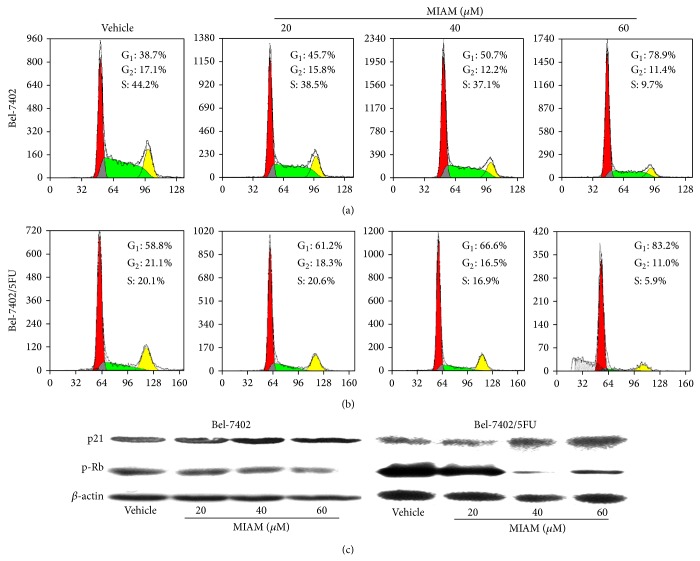
MIAM arrested cell cycles in G_0_/G_1_ phase. (a-b) Cells seeded in 6-well plate were exposed to MIAM for 72 h and then subjected to flow cytometry analysis. (c) Western blotting analyzed the expression of p21^Waf1/Cip1^ and p-Rb in HCC cells. Data are representative of 3 independent experiments.

Like many other DNA intercalator and topoisomerase inhibitors, MIAM might also exert its action by inducing DNA damage and consequently resulted in cell cycle arrest in cancer cells [20]. In the current study, we performed the flow cytometry assay to analyze the statues of cell cycle. MIAM treatment resulted cell cycle arrest in G_0_/G_1_ phase. MIAM at the range of 20 to 60 *μ*M led to an increase of cell cycle in G_1_ phase by 45.7%, 50.7%, and 78.9% (Figure 3(a), 20 *μ*M, *P* > 0.05; 40 and 60 *μ*M, *P* < 0.01 versus the vehicle control), respectively, in Bel-7402 cells, and by 61.2%, 66.6%, and 83.2% (Figure 3(b), 20 *μ*M, *P* > 0.05; 40 and 60 *μ*M, *P* < 0.01 versus the vehicle control), respectively, in Bel-7402/5FU cells.

Cell cycle transition from G_1_ to S phase is generally governed by p21^Waf1/Cip1^ and dephosphorylation of Rb. As shown in Figure 3(c), MIAM with 20, 40, and 60 *μ*M significantly increased the levels of p21^Waf1/Cip1^ in both Bel-7402 and Bel-7402/5FU cells. Moreover, the levels of p-Rb were subsequently decreased in the MIAM-treated cells. Further analysis indicated that the effects of MIAM on regulation of these proteins were more pronounced in Bel-7402/5FU cells than Bel-7402 cells (Figure 3(b), *P* < 0.05).

### 3.4. MIAM Increased the Level of Cellular ROS More Potential in Bel-7402/5FU Cells Than Bel-7402

**Figure 4:**
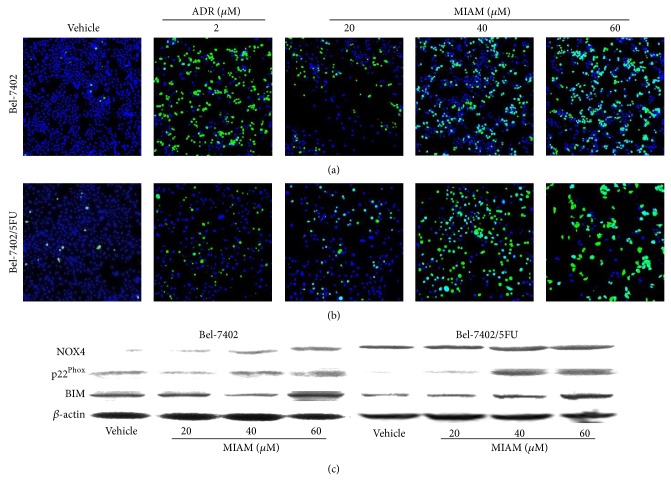
MIAM increased the level of cellular ROS in HCC cells. (a-b) Bel-7402 and Bel-7402/5FU cells were exposed to MIAM and ADR for 72 h, respectively. After staining with DCFH-DA probe, the levels of cellular ROS were visualized by ArrayScan XTI high-content imaging instrumentation. (c) Western blotting analyzed the expression of NOX4, p22^phox^, and BIM in HCC cells. Data are representative of 3 independent experiments.

DNA damage from various sources has been shown to increase reactive oxygen species (ROS) levels in cancer cells [21]. In this study, the levels of cellular ROS were increased in both Bel-7402 and Bel-7402/5FU cells exposed to MIAM at 20 to 60 *μ*M for 72 h. As shown in Figures 4(a) and 4(b), the levels of DCF fluorescence were significantly increased in Bel-7402 cells by 25.4%, 46.4%, and 52.8% (*P* < 0.05 versus the vehicle control), respectively, and in Bel-7402/5FU cells by 23.5%, 52.7%, and 76.9% (*P* < 0.05 versus the vehicle control), respectively. ADR with 2 *μ*M significantly increased the level of cellular ROS by 43% (*P* < 0.05 versus the vehicle control), whereas this concentration of ADR did not significantly increase the level of ROS in Bel-7402/5FU cells. Further, an increase of ROS in Bel-7402/5FU cells was seen by up to 20 *μ*M of ADR (Figure 4(b)).

NADPH oxidase (NOX) was originally identified as the major source of intracellular ROS in various cell types. Several studies demonstrated that NOX4 is expressed in several human tumors including HCC and involved in cellular senescence, mitochondrial apoptosis, and tumor suppression [22–24]. The catalytic center of this enzyme locates in intracellular membranes, such as endoplasmic reticulum. The activation of NOX4 only requires p22^phox^ and preferentially originates hydrogen peroxide as a product. Further, NOX4 activity has been described to be determined only by its mRNA/protein levels [25]. As shown in Figure 4(c), MIAM treatment resulted in increases of NOX4 and p22^Phox^ in both Bel-7402 and Bel-7402/5FU cells. The expression of NOX4 was significantly elevated by 1.92, 2.68, and 3.44 times (*P* < 0.01 versus the vehicle control), respectively, in Bel-7402 cells, and by 1.21, 1.35, and 1.35 times (20 *μ*M, *P* > 0.05; 40 *μ*M and 60 *μ*M, *P* < 0.05 versus the vehicle control), respectively, in Bel-7402/5FU cells. The expression of p22^Phox^ was does-dependently increased by 0.99, 1.41, and 1.44 times over that of vehicle control (20 *μ*M, *P* > 0.05; 40 *μ*M and 60 *μ*M, *P* < 0.05 versus the vehicle control), respectively, in Bel-7402 cells, and by 2.26, 3.92, and 3.81 times (*P* < 0.01 versus the vehicle control), respectively, in Bel-7402/5FU cells. These results indicated that the NOX4/p22^Phox^ pathway was activated in both Bel-7402 and Bel-7402/5FU cells. Activation of NOX4 is required for the upregulation of BIM, the proapoptotic BH3 only gene [23]. As shown in Figure 4(c), MIAM at 40 and 60 *μ*M significantly increased the levels of BIM in Bel-7402/5FU cells by 210.3% and 265.8% (*P* < 0.05 versus the vehicle control), respectively, whereas the level of BIM was only increased in Bel-7402 cells exposed to MIAM at 60 *μ*M by 178.2% (*P* < 0.05 versus the vehicle control). Bel-7402/5FU cells were demonstrated to be more sensitive to MIAM in increasing BIM than Bel-7402 cells (*P* < 0.01 versus the vehicle control). These results suggested that MIAM treatment resulted in the activation of NOX4 and subsequently the upregulation of proapoptotic BIM in HCC cells. Furthermore, these effects of MIAM were more obvious in Bel-7402/5FU cells than in Bel-7402 cells.

### 3.5. MIAM Increased Expression of SIRT3 and p53 More Significant in Bel-7402/5FU Cells Than Its Sensitive Counterpart

**Figure 5:**
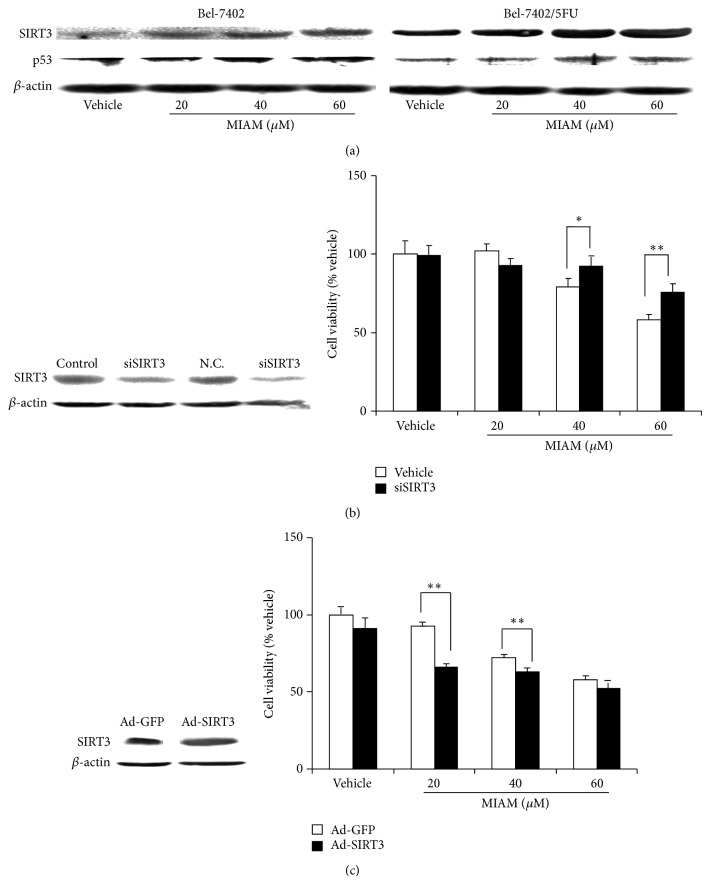
The upregulation of SIRT3 in HCC cells exposed to MIAM. (a) HCC cells were exposed to increasing concentration of MIAM for 72 h. Western blotting analyzed the expression of SIRT3 and p53 in HCC cells. Data are representative of 3 independent experiments. (b) The silencing of SIRT3 by transfection with siRNA of SIRT3 in Bel-7402/5FU cells (Figure 5(b), left). Bel-7402/5FU cells were incubated with MIAM for 48 h (Figure 5(b), right). Viable cells were estimated by the MTT assay and denoted as a percentage of untreated controls at the concurrent time point. The bars indicate mean ± S.D. (*n* = 5). (c) Overexpression of SIRT3 by transfection with human SIRT3 adenovirus (Ad-SIRT3) or control adenovirus expressing GFP (Ad-GFP) in Bel-7402/5FU cells (Figure 5(c), left); and then cells were incubated with MIAM for 48 h (Figure 5(c), right). Viable cells were estimated by the MTT assay and denoted as a percentage of untreated controls at the concurrent time point. ^∗^ *P* < 0.05, ^∗∗^ *P* < 0.01 versus the vehicle control. The bars indicate mean ± S.D. (*n* = 5).

SIRT3 is a member of mammalian sirtuin family that is localized to mitochondria and plays a crucial role in control of cell fate. Absence of SIRT3 in HCC cells was found to associate with poor response to chemotherapy in HCC patients. Moreover, SIRT3 overexpression could trigger apoptosis through activation of p53. In the present study, MIAM was found to increase the expression of SIRT3 more significant in Bel-7402/5FU cells than Bel-7402 cells. Consequently, p53, the downstream effector of SIRT3, was more significantly increased in Bel-7402/5FU cells too. As shown in Figure 5(a), MIAM at 20, 40, and 60 *μ*M significantly increased the levels of SIRT3 by 150.0%, 215.2%, and 320.3% (*P* < 0.01 versus the vehicle control), respectively, in Bel-7402 cells, and by 180.6%, 257.7%, and 352.6% (*P* < 0.01 versus the vehicle control), respectively, in Bel-7402/5FU cells. Consequently, MIAM at 20, 40, and 60 *μ*M significantly increased the levels of p53 by 1.12, 2.31, and 2.42 times, respectively, in Bel-7402 cells, and by 0.2, 4.69, and 4.51 times, respectively, in Bel-7402/5FU cells. Noteworthy, the loading amount of protein for evaluating SIRT3 levels in Bel-7402 cells was 2 times higher than that in Bel-7402/5FU cells. Therefore, it suggested that the upregulation of SIRT3 might play the central role in mediating the increase of p53 in Bel-7402/5FU cells.

To further determine the role of SIRT3 in mediating MIAM induced HCC cell death, we examined the effect of MIAM on the growth of Bel-7402/5FU after SIRT3 deletion or overexpression. MIAM did not significantly alter the status of proliferation in the SIRT3 siRNA cells, whereas the adenovirus expressing SIRT3 cells were demonstrated to be very sensitive to MIAM treatment. As shown in Figure 5(b) (left), transfection of siRNA achieved the result of silencing of SIRT3 by 80%. Using these cells, MIAM at 20, 40, and 60 *μ*M for 48 h exposure resulted in the percent of inhibition by only 9.2%, 7.9%, and 24.4%, respectively. There was a significant difference between the SIRT3 siRNA cells and Bel-7402/5FU cells (*P* < 0.05, as compared to Figure 1(b)). In Bel-7402/5FU cells with adenovirus expressing SIRT3, the level of SIRT3 was strongly increased by 257.3%. Using these cells, MIAM treatment for 48 h resulted in inhibition by 32.8%, 37.9%, and 47.8%, respectively. We therefore suggested that MIAM inhibit HCC growth more potential in SIRT3 overexpressed Bel-7402/5FU cells than SIRT3 siRNA and Bel-7402 cells. SIRT3 might be a target of MIAM in inhibition of HCC.

## 4. Conclusion

Current study presented that MIAM, a novel indole compound, inhibited HCC growth in both Bel-7402 and Bel-7402/5FU cells. MIAM could induce HCC cells to apoptosis and arrest cell cycle in G_0_/G_1_ phase. Importantly, MIAM inhibited HCC growth more potent in Bel-7402/5FU cells than the parent Bel-7402 cells. Further studies indicated that these effects of MIAM might be due to its actions in induction of cellular ROS and activation of NOX4/p22^Phox^/BIM and SIRT3/p53/p21 pathways. The activation of SIRT3/p53/p21 pathway by MIAM was more profound in Bel-7402/5FU cells than Bel-7402 cells. To investigate the mechanism of MIAM, we performed the experiments of transfection SIRT3-targeted siRNA or adenovirus expressing SIRT3 in Bel-7402/5FU cells. MIAM did not significantly inhibit the growth of SIRT3 siRNA in Bel-7402/5FU cells, whereas the Bel-7402/5FU cells with high level of SIRT3 were shown to be more sensitive to MIAM. These results suggested that the inhibitory effect of MIAM on HCC might mainly associate with its action in upregulation of SIRT3 in Bel-7402/5FU cells. Although the role of SIRT3 in downregulation of HCC growth has not been fully elucidated, these observations indicated that SIRT3 might be a target of MIAM. We suggested that MIAM could be developed as a potential agent that might effectively inhibit HCC growth.
